# Sensitivity and Specificity of Instrumentation Lab Age-Adjusted D-Dimer Threshold Values in a Single Hospital Site: A Retrospective Analysis

**DOI:** 10.7759/cureus.30719

**Published:** 2022-10-26

**Authors:** Ryan Cracknell, Ehsan Salim

**Affiliations:** 1 Cardiology, Golden Jubilee National Hospital, Glasgow, GBR; 2 Radiology, University Hospital Monklands, Glasgow, GBR

**Keywords:** hospital admission, pleuritic chest pain, pulmonary embolism, d-dimer, ctpa

## Abstract

Introduction

The D-dimer is a common test in the assessment of chest pain in acute settings. With a high sensitivity and low specificity, a significant number of false positive outcomes occur, leading to unnecessary medical intervention. There is good evidence supporting the use of an age-adjusted D-dimer model to increase diagnostic specificity in the context of a conventional “D-dimer” assay. There is, however, a lack of evidence validating the age adjustment process when considering the less common but still widely utilized “instrumentation lab” assay.

Methods

A retrospective audit was carried out in a district general hospital by obtaining all acute computed tomography pulmonary angiograms carried out between December 2020 and August 2021. The age-adjusted D-dimer was calculated for each patient by multiplying the patient's age by 5. Thereafter, sensitivity and specificity were reassessed.

Results

After exclusion, 133 patients under 50 years of age with low pre-test probability scores were included in the analysis. Age-adjusted D-dimer was found to increase specificity from 2% to 28% whilst maintaining a sensitivity of 94%.

Conclusion

Utilization of the 5x age-adjusted instrumentation lab assay D-dimer results in increased specificity with the potential to reduce the number of unnecessary admissions, radiation exposure, and medication use, improving patient safety and reducing healthcare burden.

## Introduction

Chest pain is a common presentation to emergency and acute medical departments throughout the world [1]. Despite its commonplace at the front door of the hospital, chest pain continues to be a symptom resulting in diagnostic uncertainty. Around 15% of all chest pain presentations to acute medicine are screened for pulmonary embolism (PE) [2].The diagnostic process combines skilled history-taking alongside routine and symptom-specific investigations, including pre-test probability scoring and the high-sensitivity, low-specificity D-dimer test. Following a positive D-dimer, the patient then most commonly undergoes a computed tomography pulmonary angiogram (CTPA). CTPA is the gold standard diagnostic test in the assessment of PE with a high positive and high negative sensitivity and specificity diagnostic value [3]. Despite its high diagnostic value, the CTPA has its disadvantages, such as radiation and contrast medium exposure, expense, and time consumption/use of already constrained resources [4]. Considering the negative factors associated with CTPAs, their use should be limited where possible. The D-dimer test acts as a gatekeeper to CT scanning. If the D-dimer is negative and pre-test scoring identifies low/medium risk, PE can be excluded, without the need for imaging [5]. Despite its high negative predictive value (NPV), as a byproduct of cross-linked fibrin degradation, a raised D-dimer can be attributed to a number of prothrombotic states, including those that are physiological such as advanced age [6]. The physiological increase in D-dimer value in older adults leads to an overall reduced ability to exclude PE in this population without a CTPA [7-9].

The use of a poorly specific test in the emergency department and medical receiving wards results in unnecessary admissions, increased risk of hospital-acquired infections, cost to the health service, and exposure of patients to unnecessary radiation and anticoagulation.

A validated modification to account for physiological increases in D-dimer with age should result in increased specificity with preserved sensitivity. In this study, we add to the limited data, which suggest that adjusting the instrumentation lab D-dimer for age is valid, safe, and effective in the screening of PE.

Age-adjusted D-dimer in the context of the conventional D-dimer assay has been shown to have an increased specificity to PEs without any reduction in sensitivity [10]. The improvements demonstrated, and safety validation evidenced by conventional D-dimer age adjustment, has not been replicated when considering the less common but still widely utilized instrumentation lab assay. Therefore, due to a lack of evidence, hospitals using the instrumentation lab assay are at a disadvantage during the diagnostic work-up of PE. This impacts the patient population who attend such hospitals, as they are more likely to receive avoidable anticoagulation and undergo unnecessary CTPAs.

The aim of this study was to further evidence the validity and sensitivity of the instrumentation lab assay age-adjusted D-dimer at a single UK hospital site with the hope that hospitals in the local area, utilizing said D-dimer assay, may start to implement age adjustment in their front-door assessments of pulmonary emboli.

## Materials and methods

**Figure 1:**
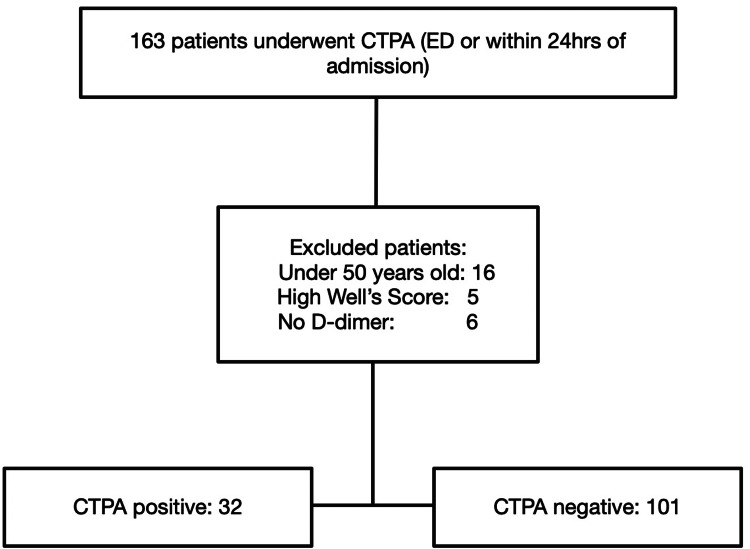
Patient recruitment, exclusion, and outcomes of CTPA scan CTPA, computed tomography pulmonary angiogram

As part of a service improvement project in a district general hospital, data were collected retrospectively for a cohort of patients who had undergone a CTPA scan as part of the diagnostic work-up for PE between December 2019 and August 2020 (n=163). The local radiology department provided a list of all patients with a CTPA requested directly from the emergency department or within the first 24 hours of admission to the medical receiving unit. Patients were excluded if they were under the age of 50 years (n=16), if they had a high Wells score (n=five), or if no D-dimer was taken on admission (n=6). The recruitment, exclusion, and outcomes of the CTPA scans have been demonstrated in Figure 1.

Following exclusion of patients for age greater than 50 years and high pre-test probability score, D-dimer results (HemosLTM assay) were recorded. The reports of the CTPA scans were either completed by or validated by consultant radiologists.

Our local laboratory used D-dimer units (HesmosLTM assay) as opposed to fibrinogen equivalent units, and thus the D-dimer result was considered positive using our locally accepted cut-off of 230 ng/L. The age-adjusted D-dimer threshold was calculated by multiplying the patient’s age by 5. The age adjustment process considered the D-dimer to be negative if it was lower than this newly calculated threshold. This negative cut-off was chosen in light of previously published data that considered age adjustment in the context of D-dimer units with a similar positive threshold [11]. This calculation is rationalized by referencing the well-evidenced conventional age-adjusted D-dimer assay where fibrinogen equivalent units (FEU) refer to a threshold of 10 times the patents age, with a standard non age-adjusted threshold of 500 nm/mL. As the instrumentation lab assay is referenced in D-dimer units with a threshold roughly half that of the D-dimer assay (230 nm/mL), it is logical to therefore half the age adjustment calculation (five times the age as opposed to 10).

Data were analyzed using SPSS Version 28.0 (IBM Corp., Armonk, NY) and Microsoft Excel Version 16.61. As a result of the sample size, the Shapiro-Wilk test was used to test normal distribution. Normally distributed values are presented as mean and standard deviation, whilst those not normally distributed are presented as median and interquartile range. Welch’s t-test was used to test normally distributed parametric variables for significance. Where not normally distributed, the Mann-Whitney U test was used. The chi-square test was used to analyze frequencies. A 95% confidence interval was used when referencing sensitivities and specificities.

## Results

From December 2020 to August 2021, a total of 163 patients underwent CTPAs in the diagnostic work-up of PE. After exclusion, 133 patients over the age of 50, with low-to-moderate Wells scores, underwent CPTAs for suspected PE. Of the included patients, 32 (24.0%) were diagnosed with PE. The confirmed PE group had significantly higher D-dimer levels than those who were negative; however, there were no significant differences in age or sex distribution.

Conventional D-dimer was found to have 99 false-positive results and 0 false-negative results, with true-positive and true-negative results of 32 and 2, respectively. This is relayed as a sensitivity of 1.00 (95% CI: 0.89-1.00) and a specificity of 0.02 (95% CI: 0.002-0.069). The positive predictive value (PPV) and NPV of the conventional D-dimer were 0.244 (95% CI: 0.237-0.247) and 1.00, respectively.

Age-adjusted D-dimer demonstrated a reduction in false-positive results (n=73) and an increase in true-negative results (n=28). The age-adjusted D-dimer resulted in a false-negative total of 2. The sensitivity was 0.94 (95% CI: 0.79-0.99), and the specificity was 0.28 (95% CI: 0.19-0.38). The PPV and NPV were 0.291 (95% CI: 0.26-0.32) and 0.93(95% CI: 0.78-0.98), respectively. Basic demographic data, alongside PE outcomes, are demonstrated in Table 1.

**Table 1:** Basic demographic data of PE screening outcomes and statistical significance PE, pulmonary embolism

	PE	No PE	Significance
Number	32	101	
Age	70.6 (95% CI: 66.7-74.5)	70.7 (95% CI: 68.6-72.8)	p=0.482
Male	51.3%	40%	p=0.138
D-dimer	1491 (646-3291)	487 (333-772)	p<0.00001

Age adjustment was also calculated with an age-adjusted cut-off of six times the patient’s age. This led to the following results:

The sensitivity was 0.90 (95% CI: 0.75-0.98) and specificity was 0.44 (95% CI: 0.34-0.54). PPV was 0.34 (95% CI: 0.29-0.38) and NPV was 0.94 (95% CI: 0.83-0.98).

## Discussion

Most published studies looking at the use of age-adjusted D-dimers in the assessment of PE have considered the conventional D-dimer assay threshold of 500 nm/mL, with age-adjusting to 10 times the age of the patient. These studies have demonstrated that when compared to conventional D-dimer thresholds, the age-adjusted D-dimer has a higher specificity without reduction in sensitivity. Despite the large body of evidence supporting the use of conventional assay age adjustment, there are limited studies in the literature that have considered age-adjusted D-dimer in the context of the instrumentation lab D-dimer assay, which has a normal value threshold of 230 nm/mL. This study aimed to fill a gap in the literature looking at age-adjusted D-dimer in the context of the instrumentation lab D-dimer assay. Considering the age-adjusted threshold is roughly half that of the conventional D-dimer assay, a cut-off of five times the patient’s age was used. Whilst we demonstrated an improvement in specificity of over 10 times when age-adjusting the D-dimer, the sensitivity was only minimally reduced by six percent to 94%. Both false-negative results pertained to CTPA-confirmed sub-segmental PEs with no segmental or lobar involvement. These findings are similar to those reported in studies by Dutton et al. and Douma et al. [11-12] . It is debated as to whether sub-segmental PEs are pathological or are simply part of the impact of physiological ageing of the lungs, disputing the need for harmful diagnostics and treatments [13]. We conducted an ad hoc analysis where we considered sub-segmental PEs as negative findings, resulting in the sensitivity of the five times age-adjusted D-dimer increasing from 94% to 100%.

A recent study by Dutton et al. considered alternative age adjustment thresholds and showed an improved specificity with only a mild reduction in sensitivity when age-adjusting by six times the patient’s age [11]. We have therefore also considered this in our data evaluation. In this study, the multiplication of the patient’s age by six rather than five increased the specificity from 27% to 44% but reduced sensitivity from 94% to 90% due to an additional false-negative result, which was a segmental PE. It could be debated as to whether such a reduction in sensitivity, in the context of a truly segmental PE, is worth the risk for such an improvement in specificity. Patient safety has to be at the forefront of any investigative method or treatment modality. We would therefore suggest that based on this study, the increased specificity comes at too high a cost, and maintaining an age adjustment of five times the age is most appropriate. The number of patients in this retrospective analysis is relatively small, and performing such analysis on a larger cohort of patients would provide a much more accurate representation of the true reduction in sensitivity.

Utilizing an age-adjusted threshold would have resulted in an additional 30 patients having PE excluded with D-dimer alone, preventing the need for a CTPA and interim anticoagulation. This would have avoided the possible harms associated with CT scanning, such as radiation exposure and contrast nephropathy (which around 14% of patients experience after a CTPA) [14]. It would also have potentially reduced the length of stay in the hospital and the associated costs with further investigations and management.

There have been some contentions in recent years as to the value of an age-adjusted D-dimer. Despite its clear ability to reduce the false-positive PE rate and its relatively safe false-negative rate, the age-adjusted D-dimer is debated. This is evident in a recent paper by Lapner et al. who suggested that the conventional D-dimer threshold should be a “mean” as opposed to age adjusted for each patient [15]. This view has been challenged by supporters of the age-adjusted strategy. Dutton et al. tested their cohort of patients against those in Lapner et al.’s study and found that the “mean” D-dimer threshold demonstrated a reduced specificity when compared to age adjustment.

Though this study has investigated the utility and safety of the age-adjusted D-dimer when considering the instrumentation lab assay, it has been evidenced across a range of other assay types. Farm et al. demonstrated similar results to this paper when looking at the use of an age-adjusted D-dimer across four different assays, evidencing an improved specificity and an only marginally, if at all, reduced sensitivity [16].

We would hope that the results of this study allow hospital sites utilizing the instrumentation lab D-dimer assay to implement an appropriate age-adjustment strategy at the front door of the hospital, increasing their ability to discharge patients without the need for further investigations or interim treatment.

This retrospective analysis has arguably provided the evidence required to implement an age-adjusted strategy in the work-up of PE when working with the instrumentation lab assay. Assuming such a strategy is implemented, real-time analysis of the anticipated improvement in patient discharge, reduction in CTPAs, and prescription of interim anticoagulation would provide further evidence for the ongoing use of such a protocol.

This is a self-limiting retrospective study that carries its own inherent limitations. The findings, however, are similar to those obtained in prospective studies using the traditional 500 ng/mL threshold. The study excludes patients with high risk scores and does not assess age-adjusted D-dimer in varying age ranges. Despite the selected population, the NPV is reassuringly high. It would be useful to perform a prospective study using the age-adjusted threshold with this D-dimer assay.

## Conclusions

Age-adjusting the D-dimer cut-off by five times the patient’s age is safe when utilized in non-high-risk patients (under 50 years of age). It is an effective approach to increasing the specificity whilst maintaining a high level of sensitivity in the clinical setting of suspected PE. The number of false-positive results is therefore reduced, which, in turn, improves service utilization by minimizing further futile investigations and pharmacological interventions. In our cohort, five times age adjustment would have resulted in 30 (23%) patients avoiding unnecessary CTPAs and interim anticoagulation.
